# Lsa21, a novel leptospiral protein binding adhesive matrix molecules and present during human infection

**DOI:** 10.1186/1471-2180-8-70

**Published:** 2008-04-29

**Authors:** Marina V Atzingen, Angela S Barbosa, Thales De Brito, Silvio A Vasconcellos, Zenáide M de Morais, Dirce MC Lima, Patricia AE Abreu, Ana LTO Nascimento

**Affiliations:** 1 Centro de Biotecnologia, Instituto Butantan, Avenida Vital Brazil, 1500, 05503-900, São Paulo, SP, Brazil; 2 Interunidades em Biotecnologia, Instituto de Ciências Biomédicas, USP, São Paulo, Brazil; 3 Instituto de Medicina Tropical, Departamento de Patologia, Faculdade de Medicina, Universidade de São Paulo, Brazil; 4 Laboratório de Zoonoses Bacterianas do VPS, Faculdade de Medicina Veterinária e Zootecnia da Universidade de São Paulo, Brazil

## Abstract

**Background:**

It has been well documented over past decades that interaction of pathogens with the extracellular matrix (ECM) plays a primary role in host cell attachment and invasion. Adherence to host tissues is mediated by surface-exposed proteins expressed by the microorganisms during infection. The mechanisms by which pathogenic leptospires invade and colonize the host remain poorly understood since few virulence factors contributing to the pathogenesis of the disease have been identified. Whole-genome sequencing analysis of *L. interrogans *allowed identification of a repertoire of putative leptospiral surface proteins.

**Results:**

Here, we report the identification and characterization of a new leptospiral protein that exhibits extracellular matrix-binding properties, called as Lsa21 (leptospiral surface adhesin, 21 kDa). Compatible with its role in adhesion, the protein was shown to be surface-exposed by indirect immunofluorescence. Attachment of Lsa21 to laminin, collagen IV, and plasma fibronectin was specific and dose dependent. Laminin oxidation by sodium metaperiodate reduced the protein-laminin interaction in a concentration-dependent manner, indicating that laminin sugar moieties are crucial for this interaction. The gene coding for Lsa21 is present in pathogenic strains belonging to the *L. interrogans *species but was not found in the saprophytic *L. biflexa *serovar Patoc strain Patoc 1. Loss of gene expression occurs upon culture attenuation of pathogenic strains. Environmental factors such as osmolarity and temperature affect Lsa21 expression at the transcriptional level. Moreover, anti-Lsa21 serum labeled liver and kidney tissues of human fatal cases of leptospirosis.

**Conclusion:**

Our data suggest a role of Lsa21 in the pathogenesis of leptospirosis.

## Background

Leptospirosis, a worldwide zoonotic infection, is an important human and veterinary health problem. Caused by spirochaetes of the genus *Leptospira*, the disease presents greater incidence in tropical and subtropical regions [1,2]. The transmission of leptospirosis has been associated with exposure of individuals near to wild or farm animals [3]. Recently, the disease has been prevalent in cities with sanitation problems and a large population of urban rodent reservoirs, which contaminate the environment through their urine [4]. In the host, leptospirosis has a biphasic clinical presentation beginning with a septicemic followed by an immune phase with antibody production and urinary excretion of leptospires. Children primarily show fever, vomiting, headache, diarrhea, abdominal and generalized muscle pain, whereas adults have fever, headache, anorexia, muscle pain and constipation [4,5]. The most severe form of leptospirosis, known as Weil's syndrome, seen in 5 to 15% of patients, is a multisystemic febrile illness, chiefly with hepatic, renal and pulmonary involvement and a mortality rate of 5 to 40% [4]. Leptospirosis also has a great economic impact in the agricultural industry because the disease affects livestock inducing abortions, stillbirths, infertility, reduced milk production and death [3,4].

The advent of whole-genome sequencing has greatly impacted on the microbial field with the development of new large-scale technologies, such as bioinformatics. This approach has the advantage of revealing proteins independently of their abundance and without the need of culturing the microorganism *in vitro *[6]. Functional genomic studies, including transcription profiles, gene cloning, protein expression and characterization complement the *in silico *analysis and help in understanding the bacterial pathogenesis. The genome of *L. interrogans *serovar Copenhageni has been sequenced and *in silico *analysis identified more than 200 predicted outer membrane proteins [7,8]. These proteins are potential targets for inducing immune responses during host infection and therefore, constitute targets for immune protection through mechanisms such as antibody-dependent phagocytosis and killing mediated by complement. In addition, it is possible that some of these membrane proteins mediate the initial adhesion to host cells [9-11]. Leptospiral adhesins have been described: a 36-kDa fibronectin-binding protein of unknown identity isolated from the outer sheath of a virulent variant of pathogenic leptospires [11], a 24-kDa laminin-binding protein named Lsa24 [9]/LfhA [12], LigA and LigB proteins [10]. More recently, Lsa24 [9]/LfhA [12] was shown to belong to a paralog family designated *Leptospira *endostatin-like proteins (Len) [13]. Some proteins of the Len family were capable to bind host fibronectin [13]. Fibronectin binding activity has been also shown with LigA and LigB proteins [10] and a domain of the LigB protein that contributes to this binding has recently been described [14].

Several predicted surface-coding sequences were selected from the genome of *L. interrogans *serovar Copenhageni and are under study in our laboratory [8,9,15]. In the present study, we focused on a novel hypothetical protein of unknown function, encoded by the gene LIC10368. The gene was cloned and the protein expressed using *E. coli *as a heterologous host system. The recombinant protein of 21 kDa was purified and its capacity to mediate attachment to various extracellular matrix (ECM) components was evaluated. We have found that this novel leptospiral protein is a surface exposed adhesin that binds strongly to laminin, fibronectin (plasma) and collagen IV. The gene coding for Lsa21 (leptospiral surface adhesin of 21 kDa) is expressed in low passage virulent Fiocruz L1-130, LPF and LO4 strains of *L. interrogans*, and is regulated by osmolarity and temperature. Anti-Lsa21 serum labeled liver and kidney tissues of human fatal cases of leptospirosis. Our data suggest a role of Lsa21 in pathogenesis and virulence.

## Results

### Cloning, expression and purification of recombinant protein

**Figure 1:**
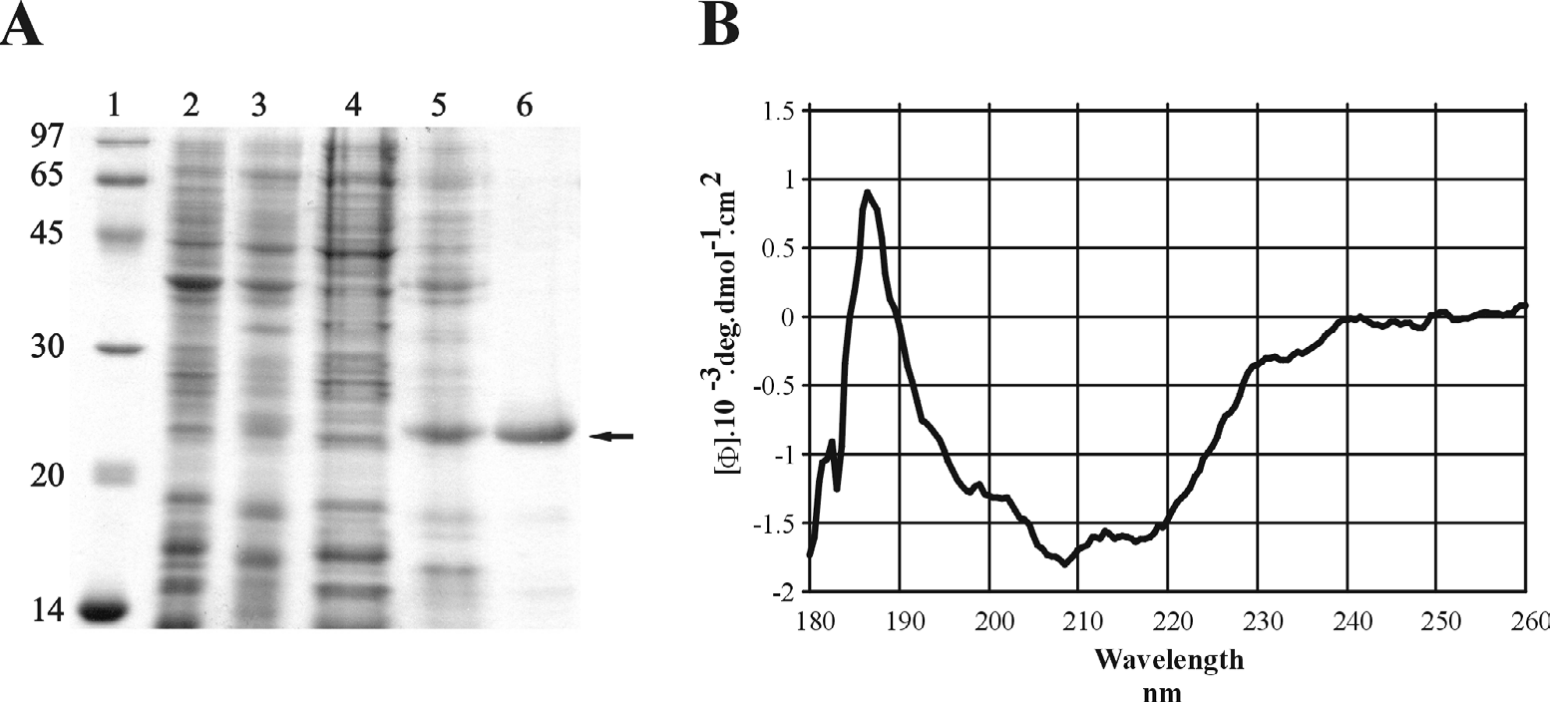
**Analysis of Lsa21 recombinant protein from NaCl-induced *E. coli *Bl21-SI by SDS-PAGE and Circular dichroism spectrum**. In **A**. SDS-15% PAGE showing lane 1 – molecular weight protein marker (kDa), lane 2 – non-induced culture, lane 3 – induced culture, lanes 4 and 5 – supernatant and inclusion body pellet after bacterial cell lysis and centrifugation, respectively, lane 6 – purified protein eluted from Ni^+2 ^– charged chelating sepharose column with 1 M imidazole. In **B**. CD spectrum of Lsa21 protein depicting both α-helical and β-sheets in its secondary structure composition. Far-UV CD spectrum is presented as an average of five scans recorded from 190 to 260 nm.

The choice of predicted surface-CDS was mostly based on cellular localization since surface proteins are potential targets for mediating adhesion to host. Thus, the LIC10368 CDS was predicted to be an outer membrane protein (93.1%) according to PSORT program [16]. The LipoP server predicted LIC10368 CDS to be a lipoprotein with a cleavage site for signal peptidase II at amino acids 18–19 [17]. It is a hypothetical protein with no known domain by BLAST [18,19] and PFAM analysis [20]. Identical predicted coding sequence of LIC10368 was identified in *L. interrogans *serovar Lai [21] but absent in *L. borgpetersenii *[22] and *L. biflexa *[23] genome sequences. The gene was amplified, without the signal peptide sequence, and the DNA insert cloned and expressed as a full-length protein in *E. coli*. Recombinant protein was expressed with 6XHis tag at the N-terminal, purified by metal chelation chromatography, and an aliquot of each step of the process was analyzed through SDS-PAGE (Fig. 1A). The expected protein band of 21 kDa is shown in NaCl-*E. coli *BL21 (SI)-induced culture, in insoluble form, as inclusion bodies (Fig. 1A, lane 5). Although the Lsa21 protein presented low expression level, it was consistently recovered from the column as single major band (Fig. 1A, lane 6). Structural integrity of the purified protein was assessed by circular dichroism (CD) spectroscopy. As depicted in Fig. 1B, the recombinant protein may encompass a mixture of both α-helices and β-sheets in its secondary structure composition. The experimental data obtained with Lsa21 is similar to the secondary structure content of the native protein predicted by computational analysis [24].

### Distribution and expression of LIC10368 gene among leptospire strains

**Figure 2:**
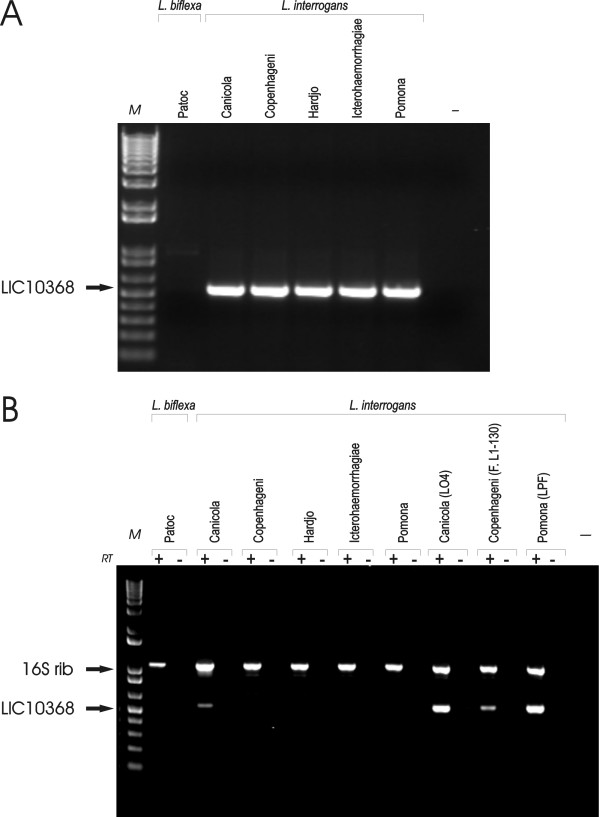
**Distribution and expression of LIC10368 gene in saprophytic and pathogenic leptospires**. **(A) **Genomic DNA from *L. biflexa *Patoc and from five serovars belonging to the pathogenic species *L. interrogans *was subjected to PCR analysis with LIC1368 specific primers designed according to *L. interrogans *serovar Copenhageni genome sequences. The expected size of the PCR product is 540 bp. No DNA was added to the negative control reaction (-). **(B) **RT-PCR analysis of LIC10368 transcripts in high-passage *L. interrogans *strains and in the low-passage LO4 (Canicola), Fiocruz L1-130 (Copenhageni), LPF (Pomona) strains. Reactions were performed with the same primer pairs mentioned above. Samples quantity and integrity were verified by amplification of a 1042-bp 16S ribosomal cDNA fragment. RT+: reverse transcriptase present; RT -: reverse transcriptase omitted; M: molecular mass markers.

The presence of LIC10368 gene in five pathogenic strains and in one saprophytic strain was examined by PCR with a pair of primers designed according to *L. interrogans *serovar Copenhageni genome sequences. A 540-bp DNA fragment covering almost the entire LIC10368 predicted CDS was amplified by PCR in all five strains belonging to the pathogenic species *L. interrogans *serovars: Canicola, Copenhageni, Hardjo, Icterohaemorrhagiae and Pomona. No amplification product was detected in the non-pathogenic *L. biflexa *serovar Patoc strain Patoc 1 (Fig. 2A). Fragments excised from the gel were purified, cloned into the pGEM-T Easy vector (Promega), and inserts were sequenced. Multiple sequence alignment of the deduced amino acid sequences of the five serovars harboring the LIC10368 gene revealed high conserved identity among the tested strains (not shown).

The ability of both high- (>200) and low- (< 3) passage *in vitro *cultured leptospires to express LIC10368 was assessed by PCR amplification of reversely transcribed total RNA. Significant levels of gene product could be detected in all three low-passage strains tested (Fig. 2B), whereas among the high-passage strains detectable amounts of LIC10368 transcripts were only observed for the Hond Utrechet IV strain (serovar Canicola). Integrity of total RNA used in RT-PCR experiments was assured by the presence of a 1,042-bp 16S ribosomal cDNA fragment in all samples (Fig. 2B).

### Regulation of LIC10368 by environmental cues

It is well known that prolonged culture promotes leptospiral virulence attenuation [25], and it has been suggested that expression of virulence factors may be downregulated upon sequential *in vitro *culture passages [26]. To evaluate the effect of culture-attenuation by *in vitro *passages on LIC10368 expression, the virulent low-passage strains *L. interrogans *serovar Pomona strain LPF, *L. interrogans *serovar Canicola strain LO4, and the reference high-passage strains *L. interrogans *serovar Pomona strain Pomona, *L. interrogans *serovar Canicola strain Hond Utrechet IV were grown to late-log phase and collected for RNA isolation. RT-PCR analyses of LIC10368 transcripts were performed, and a significant reduction of gene expression was already observed after 4 (strain LPF) or 3 (strain LO4) passages, thus indicating that LIC10368 expression is rapidly reduced with sequential *in vitro *culture passages. Amplified products were not detected in control reactions lacking reverse transcriptase, ruling out DNA contamination.

**Figure 3:**
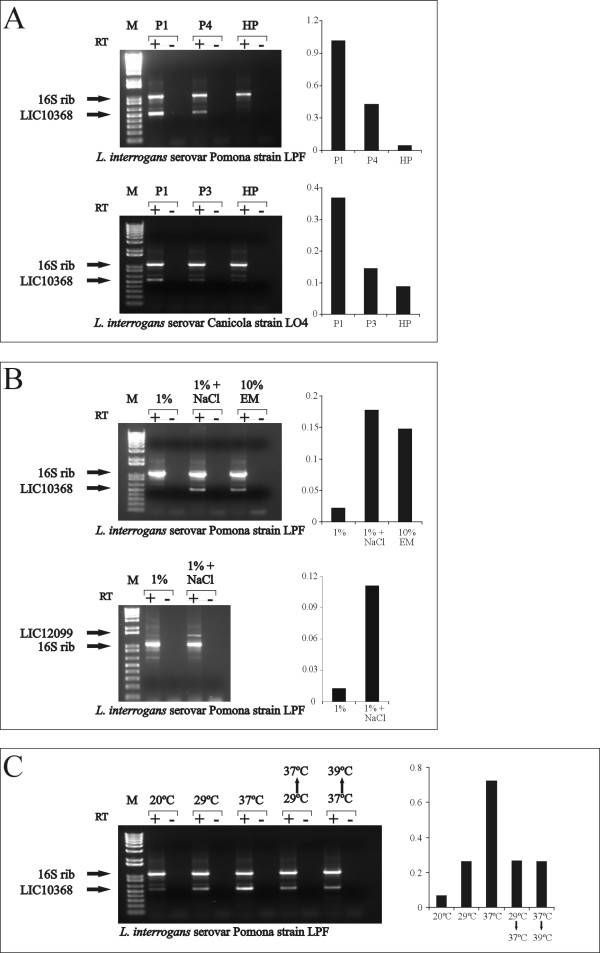
**Regulation of LIC10368 expression by environmental factors**. **(A) **RT-PCR analysis of LIC10368 transcripts upon culture-attenuation. RNA was extracted from low- and high-passage *L. interrogans *strains. Above, low-passage *L. interrogans *serovar Pomona strain LPF (passages 1 and 4, P1 and P4) and high-passage *L. interrogans *serovar Pomona strain Pomona (HP). Below, low-passage *L. interrogans *serovar Canicola strain LO4 (passages P1 and P3) and high-passage *L. interrogans *serovar Canicola strain Hond Utrechet IV (HP). **(B) **Regulation of LIC10368 transcript levels by osmolarity. Cultures of *L. interrogans *serovar Pomona strain LPF grown in EMJH with 1% rabbit serum were centrifuged and resuspended in fresh EMJH medium or in EMJH containing 120 mM NaCl. Cultures were incubated for 24 h before being harvested for RNA isolation. RNA was also obtained from a culture grown under our standard laboratory conditions (10% rabbit serum supplemented with amino acids and salts). LIC10368 transcripts obtained from cultures with 1% serum (1%), 1% serum + 120 mM NaCl (1% + NaCl) and 10% serum + amino acids and salts referred as enriched medium, EM (10% EM). 16S ribosomal cDNA fragments (1,042-bp) were co-migrated in all lanes. LIC12099 (*lipL53 *gene) transcripts were included as a positive control (1%, 1% + NaCl). **(C)** The effect of temperature on LIC10368 transcript levels was assessed by culturing leptospires at 20ºC, 29ºC and 37ºC. Additional cultures grown at 29ºC and at 37ºC were shifted overnight to 37ºC and to 39ºC, respectively, in order to simulate conditions encountered by bacteria upon entry into the host and in a febrile stage. Optical densities of LIC10368 and LIC12099 transcripts were normalized for each sample with corresponding 16S ribosomal densitometry data to obtain the relative expression levels. RT+: reverse transcriptase present in the reaction; RT-: reverse transcriptase omitted; M: molecular mass markers.

As our results pointed to a correlation between virulence and LIC10368 expression, we decided to examine whether environmental factors, such as osmolarity and temperature could influence LIC10368 regulation at the transcriptional level. All RT-PCR assays were performed with the virulent, low-passage *L. interrogans *serovar Pomona strain LPF. Induction of LIC10368 expression by osmolarity was assessed by growing cultures at 29°C in EMJH supplemented with 1% rabbit serum and resuspending them in fresh EMJH medium or in EMJH containing 120 mM NaCl. The addition of 120 mM NaCl to the medium mimics physiological conditions (~300 mosmol per liter) encountered by leptospires upon entry into the host [26]. Cultures were incubated for 24 h and examined for gene expression. LIC10368 expression was also evaluated in a culture grown under our standard laboratory conditions. As a positive control, we included RT-PCR data for the *lipL53 *gene (LIC12099) (Fig. 3B). This gene codes for a predicted lipoprotein reactive with sera from patients with leptospirosis [15] and was shown to be strongly up-regulated by sodium chloride in a recently published paper [27]. As shown in Fig. 3B, LIC10368 expression appears to be regulated by the osmolarity of the growth medium, because transcripts were detected only upon incubation with additional NaCl, or with a rich EMJH medium containing more serum and salts. Similar up regulation was observed with *lipL53 *expression, as previously detected by microarrays analysis [27].

Temperature is another important environmental signal that may affect protein expression in bacteria. The transition of temperatures from ambient to mammalian body (37° and later 39°C or higher during the febrile stage) has been correlated with changes in the expression of virulence determinants in many pathogens [28]. Moreover, it has been demonstrated that *L. interrogans *also regulates protein synthesis in response to *in vitro *temperature changes [29,30]. Therefore, we compared LIC10368 gene expression patterns of cultures grown at 20°C, 29°C and 37°C, reflecting ambient temperatures in the environment, growth under laboratory conditions, and mammalian host body core temperature. Transcriptional analyses were also performed with cultures subjected to temperature upshifts from 29°C to 37°C and from 37°C to 39°C during an overnight period to simulate conditions experienced by leptospires in the early stages of infection and during febrile stage. LIC10368 transcript levels gradually increased when cultures grown at 20°C, 29°C and 37°C were compared (Fig. 3C). An overnight upshift from 29°C to 37°C had no effect on LIC10368 expression, while an upshift from 37° to 39°C seems to have down-regulated gene expression (Fig. 3C). Our results indicate that temperature is another critical parameter involved in the control of LIC10368 gene expression. Densitometric readings provided information regarding accumulation of LIC10368 transcripts (Fig. 3A, B, C).

### Cellular localization of Lsa21 protein

**Figure 4:**
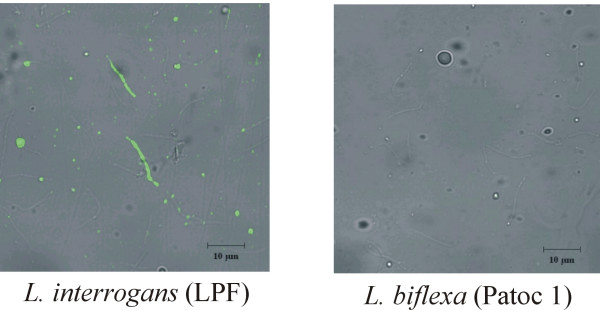
**Indirect immunofluorescence staining of intact fixed leptospires**. *L. interrogans *serovar Pomona strain LPF and *L. biflexa *serovar Patoc strain Patoc 1 were incubated with mouse anti-Lsa21 serum, followed by incubation with a fluorescein isothiocyanate-conjugated goat anti-mouse antibody. Magnification × 1,260.

Indirect immunofluorescence was used to examine whether Lsa21 protein is surface-exposed. A similar protocol has been previously employed to evaluate the capability of *Leptospira *to bind factor H [12]. The low-passage *L. interrogans *serovar Pomona strain LPF reacted with antiserum directed against recombinant Lsa21 protein, but no staining was observed with the *L. biflexa *serovar Patoc strain Patoc 1 (Fig. 4). Pre-immune serum failed to elicit a positive signal in both strains (data not shown).

### Adhesion of Lsa21 protein to ECM components

**Figure 5:**
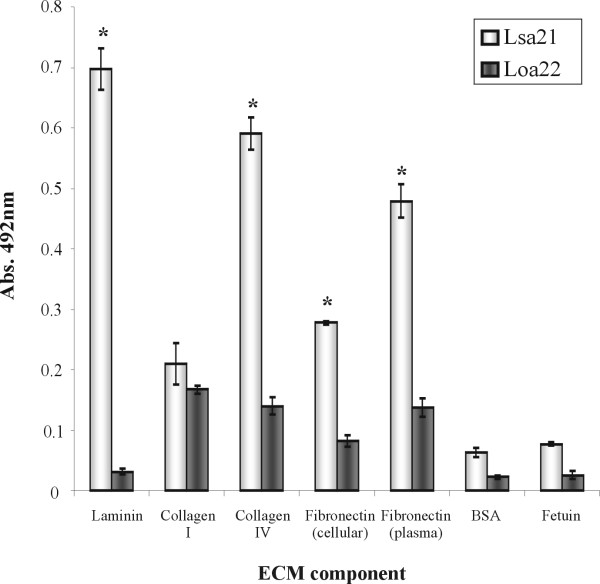
**Binding of Lsa21 recombinant protein to ECM components**. Wells were coated with 1 μg of laminin, collagen Type I, collagen Type IV, cellular fibronectin, plasma fibronectin, and the control proteins BSA and fetuin. Recombinant Lsa21 and Loa22 proteins attachment to those ECM macromolecules was assessed by an ELISA-based assay. One microgram of recombinant protein was added per well. Optical densities were taken at 492 nm. Data represent the mean ± standard error of three independent experiments. For statistical analysis, the attachment of Lsa21 to ECM macromolecules was compared to its binding to BSA by the Student's two tailed *t*- test (*, *P *< 0.01).

**Figure 6:**
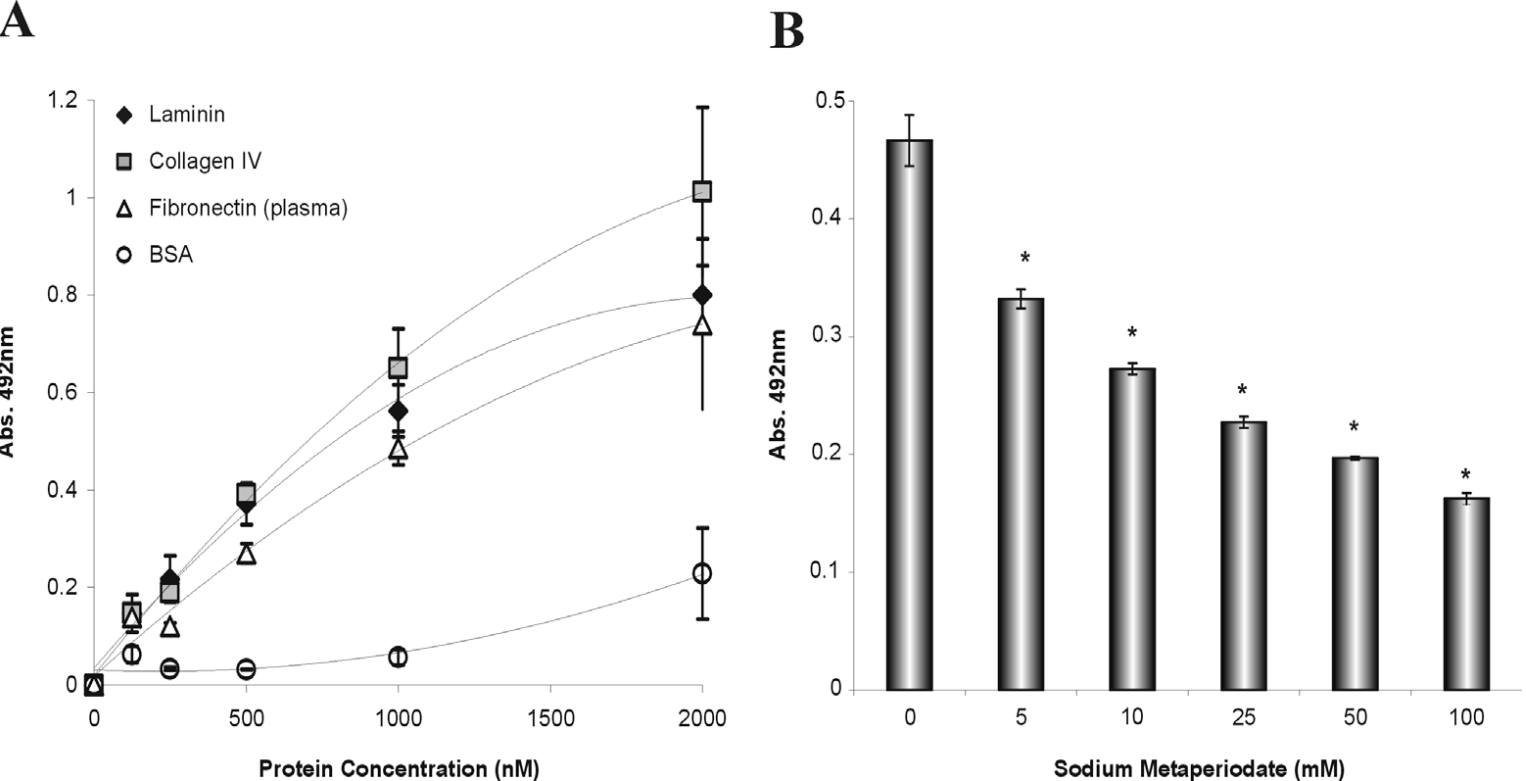
**(A) Lsa21 binds to ECM components in a dose-dependent manner**. Binding of Lsa21 to laminin, collagen IV and plasma fibronectin, as a function of protein concentration (0 to 2,000 nM). Each point represents the mean absorbance value at 492 nm ± the standard deviation of three independent experiments. BSA was included as a negative control. **(B) Sugar moiety contribution to the laminin-Lsa21 interaction**. Immobilized laminin was treated with sodium metaperiodate (5 to 100 mM) for 15 min at 4°C in the dark. Each point represents the mean absorbance value at 492 nm ± the standard deviation of three independent experiments. Reduction in attachment compared to the level of attachment to untreated laminin was statistically significant: in each case (*), the *P *value, as measured by the Student two-tailed *t *-test, was ≤ 0.004.

As Lsa21 protein is suggested to be surface-exposed and might play a role in virulence, we queried whether it could mediate host colonization by adhering to extracellular matrix proteins. Therefore, we examined its interaction with laminin, collagen type I, collagen type IV, cellular fibronectin, and plasma fibronectin. BSA and fetuin were included as negative controls and binding assays were performed by an ELISA-based assay [9]. As shown in Fig. 5, Lsa21 protein bound to all immobilized ECM macromolecules tested, but no interaction was detected with BSA or fetuin. As a negative control, we have included another recombinant protein, rLIC10191, known as Loa22 [31], and recently described as a virulence factor in *Leptospira *species [32]. A stronger interaction was observed with laminin, collagen IV and plasma fibronectin. This interaction was also assessed on a quantitative basis as illustrated in Fig. 6A. A dose-dependent binding was observed when increasing concentrations of the recombinant protein (0 to 2,000 nM) were allowed to adhere to a fixed laminin, collagen IV or fibronectin concentration (1 μg).

### Protein binding is affected by laminin oxidation

To investigate the role of laminin carbohydrate moieties in the interaction with Lsa21 protein, laminin was oxidized by increasing concentrations of sodium metaperiodate (5 to 100 mM) for 15 min at 4°C. The mild treatment ensures cleavage of vicinal carbohydrate hydroxyl groups, but the polypeptide chain structure remains intact [33]. Oxidation effect was dose-dependent and a significant reduction (~65%) in Lsa21 protein attachment to metaperiodate-treated laminin was observed at a 100 mM concentration of periodate (Fig. 6B). These data indicate that laminin sugar residues are critical for the interaction of Lsa21 protein with this major ECM glycoprotein.

### Sequence comparison between Lsa21 and leptospiral adhesions

**Figure 7:**
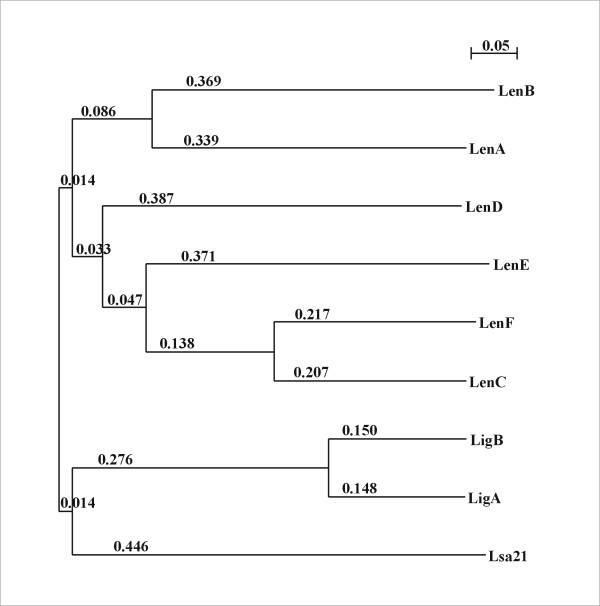
**Sequence comparison between Lsa21 and leptospiral proteins**. Unrooted phylogenetic tree of predicted amino acid sequences of the Len family, LigA/LigB and Lsa21 proteins. The tree was generated by Clustal X program and displayed by NJ plot. Branch lengths are depicted.

To investigate whether Lsa21 protein shared a sequence similarity or a common domain with other previously identified leptospiral ECM- binding proteins [9,10,13] we proceeded with a sequence analysis using Clustal X program and a tree-display NJ plot [34,35]. The calculated tree derived from sequence alignment is depicted in Fig. 7 and clearly shows that Lsa21 sequence is unrelated to both Len protein family and LigA/LigB proteins. Contrasting with the Len and Lig proteins that divide a common branch inside their family, Lsa21 appears alone on its branch.

### Lsa21 is expressed during human leptospiral infection

**Figure 8:**
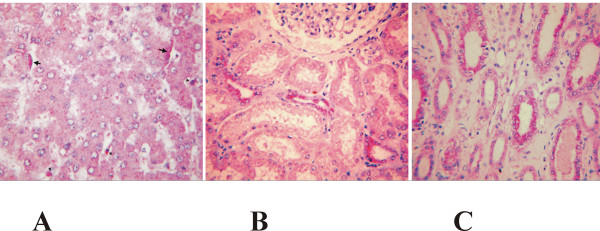
**(A) Leptospirosis, human liver**: Lsa21 is present as red deposits in the cytoplasm of isolated macrophages along the sinusoidal lining (arrow heads). Similar deposits are observed focally on the apical sinusoidal plasma membrane of hepatocytes and as small granules in the underneath cytoplasm (arrows). IHC and light hematoxylin counterstain; **(B) Leptospirosis, human kidney**: Lsa21 antigen is present over the luminal side of distal tubules. Red granules are also seen in the cell cytoplasm below. Notice the absence of Lsa21 antigen in the proximal tubules. Glomerulus present is apparently normal. IHC and light hematoxylin counterstain; **(C) Leptospirosis, human kidney**: Lsa21 antigen is present at the luminal side of collecting ducts as red granules over the epithelial cells and in the cell cytoplasm below; IHC and light hematoxylin counterstain. In **A**, **B**, and **C**: original magnification ×300.

IHC (immunohistochemistry) analysis showed essentially similar results in all 5 patients diagnosed with Weil's syndrome. It is necessary to point out that, due to the relatively short interval between autopsy and the patients' death, tissues were better preserved, and classical findings such as liver-plate disarray, although present, were not prominent. Leptospiral Lsa21 antigen was frequently detected as red deposits in the cytoplasm of isolated macrophages along the sinusoidal lining (Kupffer cells and circulating macrophages) (Fig. 7A). Similar deposits were rarely observed in macrophages of the portal inflammatory infiltrate. Lsa21 antigen was also observed focally on the sinusoidal side of the membrane of hepatocytes and as small granules in the underneath cytoplasm (Fig. 7A). Lsa21 protein was infrequently seen over endothelial cells of portal vessels and supra-hepatic branches. In the kidney Lsa21 antigen was present in the luminal side of epithelial cells of the distal nephron, particularly distal tubules and collecting ducts (Fig. 7B, 7C). As observed in liver cells, antigen granules were also identified in the cytoplasm below the cellular membrane. No definite Lsa21 expression was detected in epithelial cells of the proximal tubules. Lsa21 deposit in the cytoplasm of macrophages of the focal interstitial inflammatory infiltrate was rarely observed (see Fig. 8).

## Discussion

The characterization of leptospiral outer membrane proteins represents an important step toward the understanding of leptospirosis pathogenicity. To date, there has been little functional analysis of *Leptospira *membrane proteins, in spite of their unquestionable relevance to host-pathogen interactions. Bioinformatic analysis of the genome sequences of *L. interrogans *serovar Copenhageni revealed more than 200 predicted outer membrane proteins that merit further studies [7,8]. In this report, we have characterized one of those hypothetical proteins, a surface leptospiral adhesin of 21 kDa, named Lsa21 that may play a role in pathogenesis.

The LIC10368 expression is mostly detected in low-passage virulent strains (Figs. 2B, 3A). Culture attenuation has also been reported for the LigA and B proteins, leptospiral antigens recognized during the acute host infection [26,36]. Although a larger sample set will be required for definite conclusion, our findings suggest a correlation between Lsa21 protein and virulence.

The LIC10368 gene is up regulated by osmolarity, another distinguishing feature shared with *ligA *and *B *genes [26]. The addition of 120mM NaCl to the culture medium reproduces the host's serum osmolarity (~300 mosM), thus providing a more physiological environment for leptospiral growth. In fact, EMJH-salt supplementation enhanced Lsa21 expression (Fig. 3B). It is worth mentioning that several predicted outer membrane proteins currently under study in our laboratory are responsive to physiologic osmolarity (unpublished data). It is not surprising because 6% of the *L. interrogans *genes are susceptible to osmoregulation [27]. Osmotic control of gene expression has been reported as an environmental cue associated with virulence in a variety of pathogens [28]. In *V. cholerae*, optimum expression of cholera toxin and *Tcp pilli *occurs within an osmolarity range that probably represents that of mucosal secretions [37]. Transcription levels of *invA*, an *S. Typhimurium *invasion gene, have also been reported to be considerably higher on media with high osmolarity [38]. Moreover, genes controlling the *P. aeruginosa *capsule, an important *Pseudomonas *virulence factor, are also subjected to osmoregulation [39].

Temperature induction of LIC10368 expression was also observed (Fig. 3C). Maximal mRNA levels were detected at 37°C, the body core temperature of most mammalian species. Intriguingly, LIC10368 is not represented among the leptospiral genes found to be up-regulated in response to temperature or physiologic osmolarity, as assessed by whole-genome microarrays [27,29]. This could be explained by the fact that LIC10368 expression rapidly decreases after three or four passages in culture medium (Fig. 3A). In addition, optimum expression might occur when both physiological parameters (mammalian body core temperature and host's serum osmolarity) are combined.

Lsa21 protein exhibits extracellular matrix-binding properties. It is thus possible that it may play a role in the attachment to host tissues. Several leptospiral adhesins have been described, including a 36-kDa fibronectin-binding protein [11], a 24-kDa laminin-binding protein named Lsa24 [9]/LfhA [12], LigA and LigB proteins [10]. Recently, it has been demonstrated that *L. interrogans *contain five additional paralogs of Lsa24 [9]/LfhA [12], designated as Len-like proteins [13]. The Len proteins were all found to bind laminin and in addition LenB, LenC, LenD, LenE and LenF have shown affinity to bind host fibronectin [13].

Lsa21 protein exhibits a broader spectrum binding profile because it interacts with laminin, collagen IV and fibronectin. Similarly, other adhesins, namely the Lig proteins [10], have been reported to bind to different ECM macromolecules. In fact, attachment of *Leptospira *to several ECM macromolecules, including laminin, collagen I, collagen IV, cellular fibronectin, and plasma fibronectin was previously shown [9]. Interestingly, Lsa21 shares neither sequence similarity nor a common domain with the Len-like and the Lig proteins. Emp, a cell surface protein of *Staphylococcus aureus*, strongly interacts with fibronectin, fibrinogen, collagen, and vitronectin [40]. The outer membrane protein YadA of *Yersinia enterocolitica *has been shown to bind to laminin, fibronectin, and several types of collagens [41-43]. *Enterococcus faecalis *adhesin Ace mediates attachment to laminin, and to collagens I and IV [44]. Finally, the *Haemophilus influenzae *Hap autotransporter protein exhibits the same binding profile of Lsa21 protein: it interacts with laminin, fibronectin and collagen IV [45]. Chemical disruption of laminin carbohydrate moieties by sodium metaperiodate caused significant reduction in the binding activity of Lsa21 protein, thus indicating the sugar moiety involvement in interactions between this recombinant protein and ECM macromolecules. Possibly Lsa21 protein interacts with a particular structural epitope shared by the ECM components mentioned above. A good candidate would be complex sugars of glycosylated proteins. During infection, injury of wall vessels exposes a repertoire of adhesive glycoproteins that constitute major targets for initial adherence of pathogens [46]. The extracellular matrix-binding properties of Lsa21 together with the data of indirect immunofluorescence suggest that this protein is surface-exposed.

The presence of Lsa21 antigen as demonstrated by IHC in cells, chiefly macrophages, of the inflammatory infiltrate both in human liver and kidney is expected because leptospirosis is an acute septicemic disease involving several organs and blood vessels. Liver-plate disarray is a pathological finding described in human and experimental leptospirosis and in the human liver, its prominence is closely related to the interval between patient's death and the autopsy, thus indicating that post-mortem changes might accentuate the lesion. In any circumstance, liver-plate disarray supports speculation that cell membrane lesion might play an important role in leptospirosis pathogenesis [47]. Although cross-reactivity between anti-Lsa21 serum and other leptospiral proteins is not excluded, immunohistochemical detection of leptospiral antigens on the sinusoidal side of human hepatocytes is an infrequent finding in human leptospirosis when standard whole bacterial polyclonal sera were used [47]. The fact that Lsa21 anti-serum stained hepatocyte membranes more often might strengthen the possible role of cell membrane lesion in the pathogenesis of the disease.

Tubulo interstitial nephritis is the main manifestation of acute renal failure in leptospirosis and the renal injury is usually associated with polyuria. Hypokalemia appear frequently with an elevated urinary fractional excretion of potassium, possibly due to proximal tubular lesions that is expressed both in human and experimental leptospirosis [47-50]. The IHC detection of the leptospiral antigenic protein Lsa21 on the epithelial membrane of the distal nephron, particularly distal tubules and collecting ducts, might be, up to a certain point, unexpected when a functional correlation is attempted. However, it is known that in a normal state, urine is concentrated because of the combined functions of Henle's loop and the collecting duct. Therefore, we might speculate that the Lsa21 deposits detected by IHC chiefly in collecting ducts might be interfering with the renal water absorption in leptospirosis, in spite of the absence of definite morphological alterations. Altogether these results strongly suggest a role of this protein in the pathogenesis of the disease because no reactivity was seen in non-leptospirosis human fatal cases.

## Conclusion

We describe a novel leptospiral surface adhesin of 21 kDa, Lsa21, shown for the first time in human fatal cases of the disease, a finding that should contribute to our understanding of the molecular mechanisms of leptospiral pathogenesis.

## Methods

### *Leptospira *strains and culture conditions

The non-pathogenic *L. biflexa *serovar Patoc strain Patoc 1, the high-passage *L. interrogans *serovar Canicola reference strains Hond Utrechet IV, *L. interrogans *serovar Copenhageni strain M-20, *L. interrogans *serovar Hardjo strain Hardjoprajtino, *L. interrogans *serovar Icterohaemorrhagiae strain RGA, *L. interrogans *serovar Pomona, the virulent strains of *L. interrogans *serovar Pomona strain LPF, *L. interrogans *serovar Canicola strain LO4 and *L. interrogans *serovar Copenhageni strain Fiocruz L1-130 were cultured at 29°C under aerobic conditions in liquid EMJH medium (Difco^®^- USA) with 10% rabbit serum, enriched with L-asparagine (wt/vol: 0.015%), sodium pyruvate (wt/vol: 0.001%), calcium chloride (wt/vol: 0,001%), magnesium chloride (wt/vol: 0.001%), peptone (wt/vol: 0.03%) and meat extract (wt/vol: 0.02%). Virulence of the *L. interrogans *serovar Pomona strain LPF, *L. interrogans *serovar Canicola LO4 and *L. interrogans *serovar Copenhageni strain Fiocruz L1-130 is maintained by iterative passages in Golden Syrian hamsters. Animals were injected with 10 to 10^5 ^leptospires inocula from low and high passage cultures. Hamsters were monitored during 21 days. No significant difference in mortality taxes was observed when animals were inoculated with "no-passage" or "3–4" passages of *Leptospira *cultures and no death was observed after animal inoculation with high passage cultures. Regulation of LIC10368 expression by environmental factors such as temperature and osmolarity was evaluated in the low-passage *L. interrogans *serovar Pomona strain LPF. The effect of temperature on LIC10368 transcript levels was assessed by culturing leptospires at 20°C, 29°C and 37°C. Additional cultures grown at 29°C and at 37°C were shifted overnight to 37°C and to 39°C, respectively, to simulate conditions encountered by bacteria upon entry into the host and in a febrile stage. Induction of LIC10368 expression by osmolarity was examined by centrifugation of cultures grown at 29°C in EMJH supplemented with 1% rabbit serum followed by resuspension in fresh EMJH medium or in EMJH containing 120 mM NaCl. Cultures were incubated for 24 h before being harvested for RNA isolation.

### *'In silico' *identification and characterization of the protein

Predicted coding sequence (CDS) LIC10368 was selected from the *L. interrogans *serovar Copenhageni genome sequences based on its cellular localization prediction by PSORT program [16]. Sequence motifs, including signal peptides, lipoprotein cleavage sites and transmembrane domains were searched using specific softwares [17-20,51,52]. Sequence analysis was performed with Clustal X [35] and a tree displayed program by the Neighbour- Joining method [34].

### Cloning, expression and purification of recombinant protein

Predicted CDS LIC10368 was amplified by the PCR from total *L. interrogans *serovar Copenhageni strain Fiocruz L1-130 genomic DNA using the primer pairs 5'GATGAAAAAAAAGAAAATGAATTGAG and 5'AACGCGATTCATAGAGAGCG. PCR product was cloned into pENTR TOPO vector (Invitrogen) followed by transfer/recombination of DNA insert into the *E. coli *expression vector pDEST17 using the LR Clonase (Invitrogen). The construct was verified by DNA sequencing with appropriate vector-specific primers. Protein expression was achieved in *E. coli *BL21 (SI) strain by the action of T7 DNA polymerase under control of the osmotically induced promoter *proU *[53]. The cells were harvested by centrifugation, the bacterial pellets resuspended in 30 ml lysis buffer (500 mM NaCl, 20 mM Tris-HCl, and 0.1% Triton X-100, pH 8.0) and lysed by French Pressure (Aminco). The insoluble fraction was washed 3 times with 30 ml of buffer (20 mM Tris-HCl, 500 mM NaCl, 1 mM β mercaptoethanol, 1 M urea and 1% Triton X-100) before solubilization with 50 ml of buffer containing 20 mM Tris-HCl, 500 mM NaCl, 5 mM β mercaptoethanol, and 6 M guanidine-Cl. Protein refolding was achieved by 500× dilution with 50 mM Tris-HCl (pH 9.0) and 500 mM NaCl. The refolded protein was then purified through metal chelating chromatography in a Sepharose fast flow column (GE Healthcare), extensively dialyzed against phosphate-buffered saline (PBS) pH 7.4, 0.1% (wt/vol) glycine solution for 24 – 48 h and fractions were analyzed in 15% SDS-PAGE.

### Circular Dichroism spectroscopy

CD spectroscopy measurements were performed at 20°C in a Jasco J-810 Spectropolarimeter (Japan Spectroscopic, Tokyo, Japan) equipped with a Peltier unit for temperature control. Far-UV CD spectra were measured using a 1 mm path length cell at 0.5 nm intervals. The spectrum is presented as an average of five scans recorded from 190 to 260 nm.

### DNA isolation and PCR analysis

*Leptospira *cultures were harvested by centrifugation at 11,500 g for 30 min and gently washed in sterile PBS twice. Genomic DNA was isolated from the pellets with a guanidine-detergent lysing solution (DNAzol^® ^Reagent, Invitrogen), according to manufacturer's instructions. A 540-bp LIC10368 DNA fragment was amplified using oligonucleotides LIC10368-F: 5'GATGAAAAAAAAGAAAATGAATTGAG and R: 5' CTTCGCAACTTGTGGATAAGG and a 1450-bp LIC12099 DNA fragment was amplified using oligonucleotides LP14-F: 5' CACCACCAATGTGTTTGGTATAGCG and LP14-R: 5' CAGCGTTTTGTGATAAAATTAAC designed according to *L. interrogans *serovar Copenhageni genome sequences (GenBank accession AE016823). PCR was performed in a reaction volume of 25 μl containing 100 ng of genomic DNA, 1 × PCR buffer (20 mM Tris-HCl (pH 8.4), 50 mM KCl), 2 mM MgCl_2_, 20 pmol of each specific primer, 200 μM of each dNTP, and 2.5 U Taq DNA Polymerase (Invitrogen). Cycling conditions were: 94°C, 5 min, followed by 35 cycles at 94°C, 50 sec, 60°C (LIC10368)/55°C (LIC12099), 50 sec, 72°C, 1 min 30 sec, and a final extension cycle of 7 min at 72°C. Amplicons were loaded on a 1.5% agarose gel for electrophoresis and visualization with ethidium bromide. Gel-purified bands (Concert Rapid Gel Extraction System – Gibco BRL, Life Technologies) were cloned into the pGEM-T Easy vector (Promega, Madison, Wis.) and the products were sequenced with the primers M13F (5^'-^GTTTTCCCAGTCACGA) and M13R (5^'-^CAGGAAACAGCTATGAC) on an ABI Prism 3730 × l sequencer (SeqWright, Houston, Tex.). Multiple sequence alignment was performed with Clustal W [54].

### RNA extraction and RT-PCR analysis

*Leptospira *cultures were harvested by centrifugation at 11,500 g for 30 min. For reverse transcription (RT)-PCR, total RNA was isolated from leptospires cultured as previously mentioned by the acid guanidinium thiocyanate phenol-chloroform method using TRIzol^® ^Reagent (Invitrogen) according to the manufacturer's recommendations. One microgram of RNA from each sample was treated with 1 U of DNAse I (Invitrogen) for 15 min at RT. DNAse I was inactivated by the addition of 1 μl of 25 mM EDTA solution followed by an incubation at 65°C for 10 min. DNAse-treated RNAs were reversely transcribed using the SuperScript™ III First-Strand Synthesis System for RT-PCR (Invitrogen). One tenth of RT products were amplified in a 25 μl reaction mix using oligonucleotides LIC10368-F/LIC10368-R or LP14-F/LP14-R as described above. In experiments designed to evaluate regulation of LIC10368 expression by environmental factors (osmolarity and temperature), 30 PCR cycles, instead of 35, were employed. Samples quantity and integrity were verified by amplification of a 1,042 bp 16S ribosomal cDNA fragment using oligomers 16S-F 5'CAAGTCAAGCGGAGTAGCAATACTCAGC and 16S-R 5'GATGGCAACATAAGGTGAGGGTTGC. Cycling conditions were: 94°C, 5 min, followed by 30 cycles at 94°C, 50 sec, 62°C, 50 sec, 72°C, 1 min 30 sec, and a final extension cycle of 7 min at 72°C. All samples were tested with and without reverse transcriptase to rule out genomic DNA contamination. PCR-amplified products were loaded onto 1.5% agarose gels for electrophoresis and visualized by ethidium bromide staining. Direct sequencing was performed to confirm the identity of RT-PCR products. Densitometric readings were taken using Image-Quant 2.1 software (Amersham Pharmacia Biotech, Piscataway, NJ). Optical densities of LIC10368 transcripts were normalized for each sample with corresponding 16S ribosomal densitometry data to obtain the relative expression levels.

### Antiserum

Ten female BALB/c mice (4–6 weeks old) were immunized subcutaneously with 10 μg of Lsa21 protein. The recombinant protein was adsorbed in 10% (vol/vol) of Alhydrogel (2% Al(OH)_3_, Biosector, formerly Superfos Biosector, Denmark), used as adjuvant. Two subsequent booster injections were given at 2-week intervals with the same protein preparation. Negative-control mice were injected with PBS. One week after each immunization, the mice were bled from the retro-orbital plexus and the pooled sera were analyzed by enzyme-linked immunosorbent assay (ELISA) for determination of antibody titers.

### Immunofluorescence

The non-pathogenic *L. biflexa *serovar Patoc strain Patoc 1 and the virulent *L. interrogans *serovar Pomona strain LPF were grown to late log phase and collected by centrifugation at 11,500 g for 30 min. The cell pellets were washed three times with phosphate-buffered saline (PBS) (pH 7.4) and resuspended in a fixing solution containing 25% glutaraldehyde, 10% formalin, and PBS (pH 7.4), essentially according to the methodology described in Verma et al. [12]. Ten microliters of 1 × 10^9 ^leptospire per ml suspensions were applied to glass slides, which were then placed on ice for 1 h. After two washes with PBS, blocking was performed using 2% (wt/vol) BSA in PBS for 1 h. Slides were washed twice with PBS and incubated with a 1:50 dilution of mouse anti-Lsa21 protein serum or with preimmune serum for 1 h at room temperature (RT) in a humidifying chamber. After three washes with PBS, slides were incubated with a 1:200 dilution of a fluorescein isothiocyanate-conjugated goat anti-mouse antibody (Sigma) for 1 h at RT. Slides were washed three times with PBS and mounting was performed in the antifading agent Mowiol (Calbiochem) with 2.5% DABCO (Sigma). A coverslip was added, and the slide was left overnight at 4°C before visualization by confocal microscopy (Carl Zeiss, Inc., Jena, Germany). Images were obtained with LSM 510 Meta software and the objective used was C-Apochromat 63×, scan zoom 2.0.

### Binding of Lsa21 protein to ECM

All macromolecules, including the control proteins fetuin and BSA, were purchased from Sigma Chemical Co. (St. Louis, Mo.). Laminin-1 and collagen Type IV were from the basement membrane of Engelbreth-Holm-Swarm mouse sarcoma, cellular fibronectin was from human foreskin fibroblasts, plasma fibronectin was from human plasma and collagen Type I was from a rat tail. Protein attachment to individual macromolecules of the extracellular matrix was analyzed according to a previously published protocol Briefly, ELISA plate wells (Nunc-Immuno Plate MaxiSorp Surface) were coated with 1 μg of laminin, collagen Type I, collagen Type IV, cellular fibronectin, plasma fibronectin, BSA (nonglycosylated attachment-negative control protein) and fetuin (highly glycosylated attachment-negative control protein) in 100 μl of PBS for 2 h at 37°C. The wells were washed three times with PBS-0.05% Tween 20 (PBST) and then blocked with 200 μl of 1% BSA for 1 h at 37°C followed by an overnight incubation at 4°C. One microgram of recombinant protein was added per well in 100 μl of PBS and protein was allowed to attach to the different substrates for 1 h 30 min at 37°C. After washing six times with PBST, bound protein was detected by adding 100 μl of a 1:5,000 dilution of mouse anti-Lsa21 serum in PBS. Incubation proceeded for 1 h and after three washes with PBST, 100 μl of a 1:5,000 dilution of horseradish peroxidase (HRP)-conjugated goat anti-mouse immunoglobulin G (IgG) in PBS were added per well for 1 h. All incubations took place at 37°C. The wells were washed three times, and *o*-phenylenediamine (0.04%) in citrate phosphate buffer (pH 5.0) plus 0.01% H_2_O_2 _was added. The reaction was allowed to proceed for 15 min and was then interrupted by the addition of 50 μl of 8 M H_2_SO_4_. The absorbance at 492 nm was determined in a microplate reader (Labsystems Uniscience, Multiskan EX). For determination of dose-dependent attachment of Lsa21 protein to laminin, collagen IV and plasma fibronectin, protein concentrations varying from 0 to 2,000 nM in PBS were used.

For statistical analyses, the attachment of Lsa21 protein to ECM macromolecules was compared to its binding to BSA by the Student's two-tailed *t *-test. A *P *value less than 0.01 was considered statistically significant.

### Metaperiodate treatment of laminin

Laminin oxidation was performed as described elsewhere [9]. Briefly, microtitre wells were coated with 1 μg of laminin in 50 mM sodium acetate buffer, pH 5.0, and incubated for 16 h at 4°C. Wells were washed three times with 50 mM sodium acetate buffer, pH 5.0, and immobilized laminin was treated with different sodium metaperiodate concentrations (5 – 100 mM) in the same buffer for 15 min at 4°C in the dark. After three washes with 50 mM sodium acetate buffer, wells were blocked with 200 μl of 1% BSA for 1 h at 37°C. Binding of Lsa21 protein (1 μg in PBS per well) to periodate-treated laminin was assessed as outlined above.

### Human samples

Five patients, three males and two females, were autopsied with a diagnosis of Weil's syndrome. The age of the four patients ranged from 20 to 29 years old and one patient was 67 years old. Illness duration averaged seven days with a sudden onset manifested by headache, malaise, muscle pains and high fever. They developed jaundice, palpable enlarged liver, acute renal failure and a hemorrhagic syndrome. Epidemiological data showed that patients had had contact with contaminated water, mainly from floods and sewage. Main clinico-epidemiological data of the patients are in Table 1. Of note, two cases (4 and 5) were part of our previous publication [47]. The illness was usually of short duration and this, associated with delayed clinical diagnosis, contributed to the lack of some important confirmatory laboratorial tests. In fatal human cases and in farm animals, diagnosis by immunohistochemistry methods are specific and also offer clues to the understanding of the pathogenesis of the disease [47]. Special care was taken in selecting autopsies with typical macro and microscopical findings described in leptospirosis and performed after an average of up to five to six hours of death. Liver and kidney samples, which are more representative of the disease, were routinely fixed in formalin, embedded in paraffin and stained hematoxylin-eosin. Liver and kidney of five non- leptospirotic patients were obtained from autopsies performed within a close post-mortem interval as compared to leptospirotic patients and used as controls. Leptospiral antigens were detected by an immunohistochemical assay using polyclonal rabbit anti-serum as previously described [47] with positive results in all five leptospirotic patients. Controls were negative.

**Table 1:** Leptospirosis: clinico-epidemiological and laboratory data

**Case number/gender/age [56]**	**Clinical and epidemiological information**	**Illness duration(days)**	**Laboratory data available(leptospirosis)**
1/m/29	Contact with contaminated water; fever, headache, vomiting, muscular pain, jaundice. Pulmonary hemorrhage. Hemodynamic and hydroelectrolytic disturbances, atrial fibrillation, acute renal failure. Hepatomegaly.	14	Seroagglutination and ELISA IgM positive
2/f/22	Contact with sewage and rats. Fever, muscular pain and respiratory failure (pulmonary hemorrhage). Jaundice. Hepatomegaly.	4	None available
3/m/29	Contact with contaminated water (floods). Fever, muscular pain and respiratory failure (pulmonary hemorrhage). Jaundice. Hepatomegaly. Acute renal failure.	6	None available
4*/m/20	Fever, muscular pain for the last 5 days, jaundice, bipalpebral edema, acute renal failure, pulmonary hemorrhage, abdominal pain and vomits, low platelet count.	5	None available
5*/f/67	Contact with rats and contaminated water, fever and muscular pain for the last 8 days. Jaundice, conjunctival hemorrhage, dyspnea and hemorrhagic sputum, acute renal failure.	8	None available

m: male; f: female; * cases previously published by De Brito et al. 2006 [47].

### Immunohistochemical assay

IHC studies were performed to assess the presence and localization of leptospiral antigens as detected by the mouse anti-Lsa21 serum (1:1,000 dilution). Kidney and liver 3 μm sections were analyzed using a standard immunohistochemistry protocol [47] with EnVision labeled polymer-AP mouse/rabbit K 4018 [55]. Antigen retrieval was performed by steamer incubation of the sections in citrate pH 6.0. Staining was completed with liquid permanent Red Chromogen K0640 [55]. All specimens were then lightly counterstained with hematoxylin. This method represents a better choice for diagnosis than the usual silver stains to detect the microorganisms particularly when we are dealing with human autopsies.

All animal studies were approved by the Ethics Committee of the Instituto Butantan (Sao Paulo, Brazil). Autopsies were performed at the Department of Pathology, University of Sao Paulo, School of Medicine (Sao Paulo, Brazil) and are routinely used for diagnosis, teaching and research purposes. All protocols used were verbally approved by the Ethics Committee of the University of Sao Paulo (São Paulo, Brazil).

## Authors' contributions

MA carried out molecular cloning, protein expression and purification. AB carried out molecular genetic studies and helped to draft the manuscript. TB designed and carried out immunohistochemical assays. SV carried out organism isolation and virulence maintenance. DL participated in the histochemical assays. ZM participated in organism isolation and experimental animal infection. PA carried out RNA isolation and immunofluorescence studies. AN selected the studied sequence, conceived of the study, and participated in its design and coordination and helped to draft the manuscript.

## References

[B1] Levett PN (2003). Usefulness of serologic analysis as a predictor of the infecting serovar in patients with severe leptospirosis. Clin Infect Dis.

[B2] Levett PN (2001). Leptospirosis. Clinical microbiology reviews.

[B3] Bharti AR, Nally JE, Ricaldi JN, Matthias MA, Diaz MM, Lovett MA, Levett PN, Gilman RH, Willig MR, Gotuzzo E (2003). Leptospirosis: a zoonotic disease of global importance. The Lancet infectious diseases.

[B4] Faine S, Adler B, Bolin C, Perolat P (1999). Leptospira and Leptospirosis.

[B5] Plank R, Dean D (2000). Overview of the epidemiology, microbiology, and pathogenesis of Leptospira spp. in humans. Microbes and infection/Institut Pasteur.

[B6] Adu-Bobie J, Capecchi B, Serruto D, Rappuoli R, Pizza M (2003). Two years into reverse vaccinology. Vaccine.

[B7] Nascimento AL, Ko AI, Martins EA, Monteiro-Vitorello CB, Ho PL, Haake DA, Verjovski-Almeida S, Hartskeerl RA, Marques MV, Oliveira MC (2004). Comparative genomics of two Leptospira interrogans serovars reveals novel insights into physiology and pathogenesis. Journal of bacteriology.

[B8] Nascimento AL, Verjovski-Almeida S, Van Sluys MA, Monteiro-Vitorello CB, Camargo LE, Digiampietri LA, Harstkeerl RA, Ho PL, Marques MV, Oliveira MC (2004). Genome features of Leptospira interrogans serovar Copenhageni. Brazilian journal of medical and biological research = Revista brasileira de pesquisas medicas e biologicas/Sociedade Brasileira de Biofisica [et al.

[B9] Barbosa AS, Abreu PA, Neves FO, Atzingen MV, Watanabe MM, Vieira ML, Morais ZM, Vasconcellos SA, Nascimento AL (2006). A newly identified leptospiral adhesin mediates attachment to laminin. Infection and immunity.

[B10] Choy HA, Kelley MM, Chen TL, Moller AK, Matsunaga J, Haake DA (2007). Physiological osmotic induction of Leptospira interrogans adhesion: LigA and LigB bind extracellular matrix proteins and fibrinogen. Infection and immunity.

[B11] Merien F, Truccolo J, Baranton G, Perolat P (2000). Identification of a 36-kDa fibronectin-binding protein expressed by a virulent variant of Leptospira interrogans serovar icterohaemorrhagiae. FEMS microbiology letters.

[B12] Verma A, Hellwage J, Artiushin S, Zipfel PF, Kraiczy P, Timoney JF, Stevenson B (2006). LfhA, a novel factor H-binding protein of Leptospira interrogans. Infection and immunity.

[B13] Stevenson B, Choy HA, Pinne M, Rotondi ML, Miller MC, Demoll E, Kraiczy P, Cooley AE, Creamer TP, Suchard MA (2007). Leptospira interrogans Endostatin-Like Outer Membrane Proteins Bind Host Fibronectin, Laminin and Regulators of Complement. PLoS ONE.

[B14] Lin YP, Chang YF (2007). A domain of the Leptospira LigB contributes to high affinity binding of fibronectin. Biochemical and biophysical research communications.

[B15] Gamberini M, Gomez RM, Atzingen MV, Martins EA, Vasconcellos SA, Romero EC, Leite LC, Ho PL, Nascimento AL (2005). Whole-genome analysis of Leptospira interrogans to identify potential vaccine candidates against leptospirosis. FEMS microbiology letters.

[B16] Nakai K, Kanehisa M (1991). Expert system for predicting protein localization sites in gram-negative bacteria. Proteins.

[B17] Juncker AS, Willenbrock H, Von Heijne G, Brunak S, Nielsen H, Krogh A (2003). Prediction of lipoprotein signal peptides in Gram-negative bacteria. Protein Sci.

[B18] Altschul SF, Gish W, Miller W, Myers EW, Lipman DJ (1990). Basic local alignment search tool. Journal of molecular biology.

[B19] Altschul SF, Madden TL, Schaffer AA, Zhang J, Zhang Z, Miller W, Lipman DJ (1997). Gapped BLAST and PSI-BLAST: a new generation of protein database search programs. Nucleic acids research.

[B20] Finn RD, Mistry J, Schuster-Bockler B, Griffiths-Jones S, Hollich V, Lassmann T, Moxon S, Marshall M, Khanna A, Durbin R (2006). Pfam: clans, web tools and services. Nucleic acids research.

[B21] Ren SX, Fu G, Jiang XG, Zeng R, Miao YG, Xu H, Zhang YX, Xiong H, Lu G, Lu LF (2003). Unique physiological and pathogenic features of Leptospira interrogans revealed by whole-genome sequencing. Nature.

[B22] Bulach DM, Zuerner RL, Wilson P, Seemann T, McGrath A, Cullen PA, Davis J, Johnson M, Kuczek E, Alt DP (2006). Genome reduction in Leptospira borgpetersenii reflects limited transmission potential. Proceedings of the National Academy of Sciences of the United States of America.

[B23] Picardeau M, Bulach DM, Bouchier C, Zuerner RL, Zidane N, Wilson PJ, Creno S, Kuczek ES, Bommezzadri S, Davis JC (2008). Genome Sequence of the Saprophyte Leptospira biflexa Provides Insights into the Evolution of Leptospira and the Pathogenesis of Leptospirosis. PLoS ONE.

[B24] Jones DT (1999). Protein secondary structure prediction based on position-specific scoring matrices. Journal of molecular biology.

[B25] Haake DA, Walker EM, Blanco DR, Bolin CA, Miller MN, Lovett MA (1991). Changes in the surface of Leptospira interrogans serovar grippotyphosa during in vitro cultivation. Infection and immunity.

[B26] Matsunaga J, Barocchi MA, Croda J, Young TA, Sanchez Y, Siqueira I, Bolin CA, Reis MG, Riley LW, Haake DA (2003). Pathogenic Leptospira species express surface-exposed proteins belonging to the bacterial immunoglobulin superfamily. Molecular microbiology.

[B27] Matsunaga J, Lo M, Bulach DM, Zuerner RL, Adler B, Haake DA (2007). Response of Leptospira interrogans to physiologic osmolarity: relevance in signaling the environment-to-host transition. Infection and immunity.

[B28] Mekalanos JJ (1992). Environmental signals controlling expression of virulence determinants in bacteria. Journal of bacteriology.

[B29] Lo M, Bulach DM, Powell DR, Haake DA, Matsunaga J, Paustian ML, Zuerner RL, Adler B (2006). Effects of temperature on gene expression patterns in Leptospira interrogans serovar Lai as assessed by whole-genome microarrays. Infection and immunity.

[B30] Nally JE, Artiushin S, Timoney JF (2001). Molecular characterization of thermoinduced immunogenic proteins Q1p42 and Hsp15 of Leptospira interrogans. Infection and immunity.

[B31] Koizumi N, Watanabe H (2003). Molecular cloning and characterization of a novel leptospiral lipoprotein with OmpA domain. FEMS microbiology letters.

[B32] Ristow P, Bourhy P, McBride FW, Figueira CP, Huerre M, Ave P, Girons IS, Ko AI, Picardeau M (2007). The OmpA-Like Protein Loa22 Is Essential for Leptospiral Virulence. PLoS Pathog.

[B33] Woodward MP, Young WW, Bloodgood RA (1985). Detection of monoclonal antibodies specific for carbohydrate epitopes using periodate oxidation. Journal of immunological methods.

[B34] Perriere G, Gouy M (1996). WWW-query: an on-line retrieval system for biological sequence banks. Biochimie.

[B35] Thompson JD, Gibson TJ, Plewniak F, Jeanmougin F, Higgins DG (1997). The CLUSTAL_X windows interface: flexible strategies for multiple sequence alignment aided by quality analysis tools. Nucleic acids research.

[B36] Nally JE, Whitelegge JP, Bassilian S, Blanco DR, Lovett MA (2007). Characterization of the outer membrane proteome of Leptospira interrogans expressed during acute lethal infection. Infection and immunity.

[B37] Miller VL, Mekalanos JJ (1988). A novel suicide vector and its use in construction of insertion mutations: osmoregulation of outer membrane proteins and virulence determinants in Vibrio cholerae requires toxR. Journal of bacteriology.

[B38] Galan JE, Curtiss R (1991). Distribution of the invA, -B, -C, and -D genes of Salmonella typhimurium among other Salmonella serovars: invA mutants of Salmonella typhi are deficient for entry into mammalian cells. Infection and immunity.

[B39] Deretic V, Dikshit R, Konyecsni WM, Chakrabarty AM, Misra TK (1989). The algR gene, which regulates mucoidy in Pseudomonas aeruginosa, belongs to a class of environmentally responsive genes. Journal of bacteriology.

[B40] Hussain M, Becker K, von Eiff C, Schrenzel J, Peters G, Herrmann M (2001). Identification and characterization of a novel 38.5-kilodalton cell surface protein of Staphylococcus aureus with extended-spectrum binding activity for extracellular matrix and plasma proteins. Journal of bacteriology.

[B41] Flugel A, Schulze-Koops H, Heesemann J, Kuhn K, Sorokin L, Burkhardt H, von der Mark K, Emmrich F (1994). Interaction of enteropathogenic Yersinia enterocolitica with complex basement membranes and the extracellular matrix proteins collagen type IV, laminin-1 and -2, and nidogen/entactin. The Journal of biological chemistry.

[B42] Schulze-Koops H, Burkhardt H, Heesemann J, von der Mark K, Emmrich F (1992). Plasmid-encoded outer membrane protein YadA mediates specific binding of enteropathogenic yersiniae to various types of collagen. Infection and immunity.

[B43] Tertti R, Skurnik M, Vartio T, Kuusela P (1992). Adhesion protein YadA of Yersinia species mediates binding of bacteria to fibronectin. Infection and immunity.

[B44] Nallapareddy SR, Qin X, Weinstock GM, Hook M, Murray BE (2000). Enterococcus faecalis adhesin, ace, mediates attachment to extracellular matrix proteins collagen type IV and laminin as well as collagen type I. Infection and immunity.

[B45] Fink DL, Buscher AZ, Green B, Fernsten P, St Geme JW (2003). The Haemophilus influenzae Hap autotransporter mediates microcolony formation and adherence to epithelial cells and extracellular matrix via binding regions in the C-terminal end of the passenger domain. Cellular microbiology.

[B46] Ljungh A, Moran AP, Wadstrom T (1996). Interactions of bacterial adhesins with extracellular matrix and plasma proteins: pathogenic implications and therapeutic possibilities. FEMS immunology and medical microbiology.

[B47] De Brito T, Menezes LF, Lima DM, Lourenco S, Silva AM, Alves VA (2006). Immunohistochemical and in situ hybridization studies of the liver and kidney in human leptospirosis. Virchows Arch.

[B48] Andrade L, Rodrigues AC, Sanches TR, Souza RB, Seguro AC (2007). Leptospirosis leads to dysregulation of sodium transporters in the kidney and lung. American journal of physiology.

[B49] Magaldi AJ, Yasuda PN, Kudo LH, Seguro AC, Rocha AS (1992). Renal involvement in leptospirosis: a pathophysiologic study. Nephron.

[B50] Yang CW, Wu MS, Pan MJ (2001). Leptospirosis renal disease. Nephrol Dial Transplant.

[B51] Bendtsen JD, Nielsen H, von Heijne G, Brunak S (2004). Improved prediction of signal peptides: SignalP 3.0. Journal of molecular biology.

[B52] Krogh A, Larsson B, von Heijne G, Sonnhammer EL (2001). Predicting transmembrane protein topology with a hidden Markov model: application to complete genomes. Journal of molecular biology.

[B53] Bhandari P, Gowrishankar J (1997). An Escherichia coli host strain useful for efficient overproduction of cloned gene products with NaCl as the inducer. Journal of bacteriology.

[B54] Higgins DG (1994). CLUSTAL V: multiple alignment of DNA and protein sequences. Methods in molecular biology (Clifton, NJ.

[B55] Makeev SM, Maramovich AS, Iaroshenko VA, Kuznetsov AP, Kondakov AA, Cherniavskii VF, Gordeev PP (2002). [Epidemiological aspects of leptospirosis in the eastern regions of the Russian Federation]. Meditsinskaia parazitologiia i parazitarnye bolezni.

[B56] Egan J, Yearsley D (1986). A serological survey of leptospiral infection in horses in the Republic of Ireland. The Veterinary record.

